# Homology and enzymatic requirements of microhomology-dependent alternative end joining

**DOI:** 10.1038/cddis.2015.58

**Published:** 2015-03-19

**Authors:** S Sharma, S M Javadekar, M Pandey, M Srivastava, R Kumari, S C Raghavan

**Affiliations:** 1 Department of Biochemistry, Indian Institute of Science, Bangalore 560012, India

## Abstract

Nonhomologous DNA end joining (NHEJ) is one of the major double-strand break (DSB) repair pathways in higher eukaryotes. Recently, it has been shown that alternative NHEJ (A-NHEJ) occurs in the absence of classical NHEJ and is implicated in chromosomal translocations leading to cancer. In the present study, we have developed a novel biochemical assay system utilizing DSBs flanked by varying lengths of microhomology to study microhomology-mediated alternative end joining (MMEJ). We show that MMEJ can operate in normal cells, when microhomology is present, irrespective of occurrence of robust classical NHEJ. Length of the microhomology determines the efficiency of MMEJ, 5 nt being obligatory. Using this biochemical approach, we show that products obtained are due to MMEJ, which is dependent on MRE11, NBS1, LIGASE III, XRCC1, FEN1 and PARP1. Thus, we define the enzymatic machinery and microhomology requirements of alternative NHEJ using a well-defined biochemical system.

DNA double-strand breaks (DSBs) are the most deleterious to the genome among various lesions. Nonhomologous end joining (NHEJ) is one of the major DSB repair pathways in higher eukaryotes.^1, 2, 3^ In the absence of key NHEJ factors, another distinct but error-prone pathway known as alternative NHEJ (A-NHEJ) has been described to have an important role in DSB repair.^4, 5, 6, 7^ It has been shown that majority of A-NHEJ-mediated repair of DSBs utilize distinct microhomology regions, hence termed microhomology-mediated end joining (MMEJ).^4, 8, 9^

A-NHEJ has been proposed as a possible cause for chromosomal translocations. Studies have shown co-amplification of *c-MYC* and *IgH* locus from pro-B lymphomas in mice deficient for p53 and NHEJ.^10^ A reduced level of class switch recombination (CSR) and increased number of chromosomal rearrangements at *IgH* locus have been shown in XRCC4- and LIGASE IV-deficient murine B cells.^8^ The occurrence of robust alternative end joining has been reported in the absence of NHEJ proteins, when murine RAG proteins were absent.^11^

Unraveling the enzymatic machinery involved in alternative end joining is currently an active area of research. Recently, it was shown that MRE11-RAD50-NBS1 complex may be involved in a subset of alternative NHEJ,^5, 12, 13, 14^ whereas ATM has a regulatory role.^15^ Role of PARP1 in repairing switch regions through a microhomology-mediated pathway leading to *IgH/c-MYC* translocations during immunoglobulin CSR has been described.^16^ Besides, studies have also suggested a role for DNA LIGASE III*α* and WRN in A-NHEJ.^17^ Interestingly, XRCC1 was shown to be dispensable in A-NHEJ during CSR, whereas functional relevance of Ligase I, III and Pol *λ* have been established.^18, 19, 20^ Hence, it can be concluded that canonical NHEJ (C-NHEJ) requires LIGASE IV–XRCC4 complex, while A-NHEJ is predominant in the absence of C-NHEJ proteins and is mainly characterized by joining utilizing microhomology (MMEJ). Further, it has been demonstrated that RPA, when bound to single-stranded DNA can antagonize MMEJ.^21^ Very recently, a genetic system was reported in budding yeast to detect microhomology-mediated repair.^22^ However, little is known whether alternative NHEJ can be operative when classical NHEJ machinery is intact.^23^ A recent study suggested that MMEJ is also functional in normal mammalian cells. Besides, HR and MMEJ share the initial steps of end resection for DSB repair in mammalian cells.^24^ However, it appears that there is not much consensus among different research groups over its presence and relevance in normal cells.^23^ Therefore, several aspects of alternative NHEJ still need to be resolved. For example, its precise mechanism and microhomology length requirements are yet to be fully uncovered. Its occurrence in normal cells needs to be proved beyond doubt. Although there are independent studies showing the role of multiple proteins using gene knockdown or knockout strategies, their involvement needs to be confirmed.

In the present study, we have established a cell-free repair assay system using which we show that MMEJ is operative even in the presence of classical NHEJ machinery. Further, our data suggest that MMEJ operates not only in cancer cells but also in normal cells. We show that a minimum of 5 nt microhomology is required for MMEJ and is independent of classical NHEJ proteins such as KU70, KU80 and LIGASE IV. Finally, we show that MRN complex, XRCC1, FEN1, PARP1 and LIGASE III are the factors responsible for joining mediated through microhomology.

## Results

### A cell-free system to study backup NHEJ pathway

Biochemical assay systems are used as ideal tools for characterizing DNA repair pathways. In the present study, we have used an *in vitro* approach utilizing a cell-free system to biochemically characterize the mechanism of microhomology-mediated alternative NHEJ.

**Figure 1:**
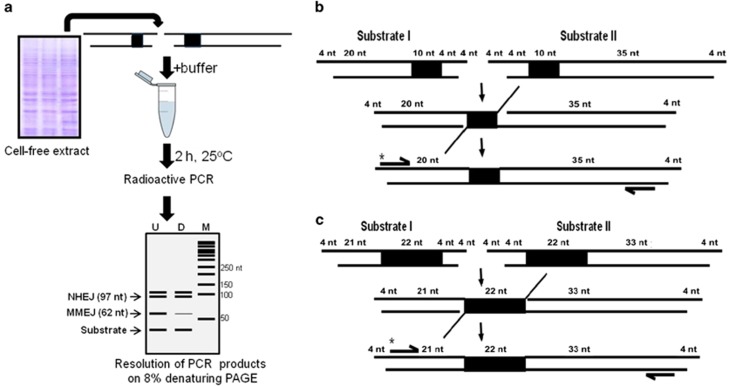
Schematic presentation of cell-free repair assay to evaluate microhomology-mediated alternative DNA end joining. (**a**) Outline of experimental strategy used for detection of MMEJ. Two double-stranded oligomers possessing 10 nt microhomology were incubated with buffer and cell-free extracts. After heat inactivaction, end-joined products were subjected to radioactive PCR and the products were resolved on 8% denaturing polyacrylamide gel. (**b** and **c)** Schematic showing strategy used for detection of alternative NHEJ products using radiolabelled oligomers. Examples of 10 nt (**b**) and 22 nt (**c**) microhomology-bearing DNA substrates are depicted to show strategy employed for detection of MMEJ. Dark rectangles indicate positions of microhomology. PCR primer positions are also indicated

**Figure 2:**
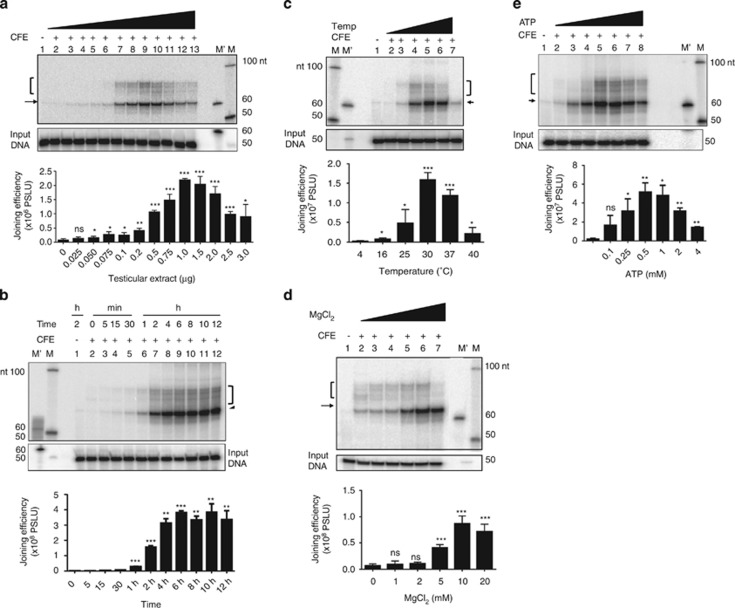
Evaluation of different experimental conditions for the development of a cell-free repair system to assess microhomology-mediated alternative DNA end joining. (**a**) Evaluation of MMEJ in presence of increasing concentrations of cell-free extracts. Testicular extracts (0, 0.025, 0.050, 0.075, 0.1, 0.2, 0.5, 0.75, 1.0, 1.5, 2.0, 2.5 and 3.0 *μ*g) were incubated with DNA substrates (4 nM) 10 nt microhomology for 2 h at 30 °C. Lane 1 indicates no protein control. (**b**) Time kinetics of MMEJ on a 10-nt microhomology-containing DNA substrates. Rat testicular extracts (1 *μ*g) were incubated with DNA substrates for 0, 5, 15 and 30 min, and 1, 2, 4, 6, 8, 10 and 12 h, and products were analyzed on 8% denaturing PAGE. (**c**) MMEJ assay at increasing incubation temperatures. A unit of 1.0 *μ*g of extract was incubated with 10 nt microhomology-containing DNA substrates in rat testicular extracts for 2 h at 4, 16, 25, 30, 37 and 40 °C. Lane 1 is no protein control. (**d**) Effect of MgCl_2_ on MMEJ catalyzed by cell-free extracts. Rat testicular extracts (1 *μ*g) were incubated with microhomology substrates and increasing concentrations of MgCl_2_ at 30 °C. Lane 1 is no protein control. Lanes 2–7 indicate MMEJ in the presence of 0, 1, 2, 5, 10 and 20 mM of MgCl_2_, respectively. (**e**) Effect of ATP on MMEJ catalyzed by cell-free extracts. Rat testicular extracts (1 *μ*g) was incubated with microhomology substrates and increasing concentrations of ATP for 2 h. Lanes 2–8 indicate MMEJ in the presence of 0, 0.1, 0.25, 0.5, 1, 2 and 4 mM of ATP, respectively. In panels **a**–**e**, bar diagram showing quantification based on at least three independent experiments are provided. MMEJ products are indicated by an arrow, while C-NHEJ products are bracketed. In each case, lower panel serves as the loading control for equal DNA, indicated as ‘input DNA'. M' and M indicate 60 nt marker and 50 nt ladder, respectively. PSLU in *y* axis of bar diagram indicates photostimulated luminescence units.*P<0.05; **P<0.01; ***P<0.001. Error bars represent S.E.M.

The steps involved in assaying MMEJ are outlined (Figure 1a). Two double-stranded oligomers of different lengths, containing 10 (or 22) nt microhomology were designed such that joining by utilizing microhomology results in shortening of the product (62 or 76 nt), which can be detected using radioactive PCR (Figures 1b and c). Similarly, joining by C-NHEJ could result in reaction products of varying sizes. In order to optimize assay conditions for MMEJ, various parameters were tested. Increasing concentrations of rat testicular extracts were incubated with DNA substrates containing 10 nt microhomology for 2 h, heat inactivated, PCR amplified using radiolabelled primers and resolved on 8% denaturing polyacrylamide gel. Results showed efficient MMEJ (62 nt) from 200 ng protein onwards, whereas C-NHEJ (~97 nt) was detectable only at protein concentration of 500 ng onwards (Figure 2a). Time kinetics analysis showed MMEJ and C-NHEJ from 5 min onwards. Interestingly, kinetics of C-NHEJ was faster compared with MMEJ (Figure 2b). Besides, testicular extracts showed optimum MMEJ at 30 ºC, the physiological temperature (Figure 2c). An enhancement in the efficiency of MMEJ was observed with an increase in concentrations of Mg^2+^, a co-factor, optimum being 10 mM (Figure 2d). Efficiency of MMEJ was maximum at 0.5 mM adenosine triphosphate (ATP; Figure 2e). Sensitivity of MMEJ assay was determined by serially diluting DNA and detecting the joining from 100 pM onwards, which exponentially increased till 4 nM (Supplementary Figure 1). Thus, we were successful in establishing a cell-free repair assay system to study MMEJ.

### MMEJ in normal and cancer cells

**Figure 3:**
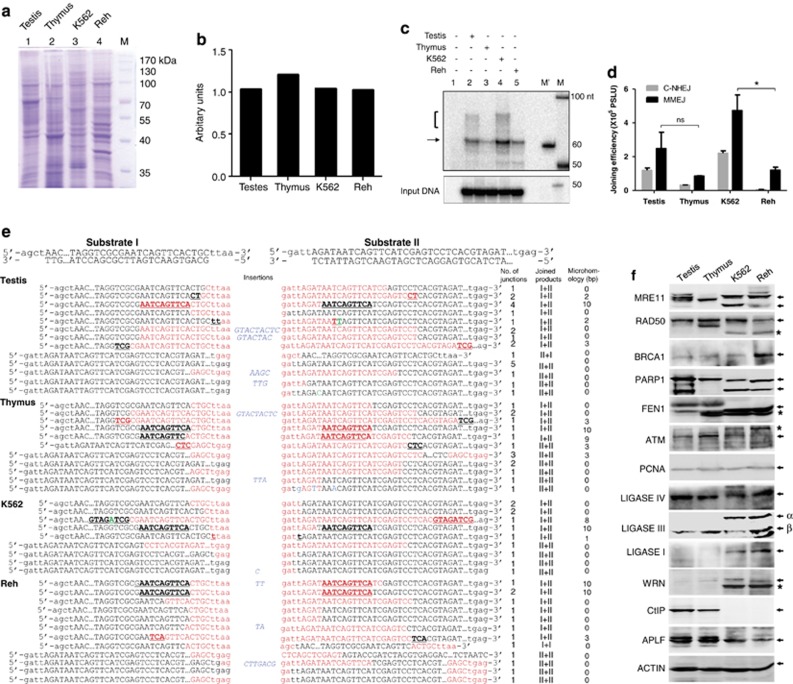
Comparison of frequency and mechanism of MMEJ between normal and cancer cells. (**a**) SDS-PAGE profile of cell-free extracts prepared from testis, thymus, K562 and Reh. (**b**) Bar diagram showing densitometry analysis for proteins in PAGE shown in panel **a**. (**c**) Comparison of MMEJ and C-NHEJ efficiency catalyzed by testis, thymus, K562 and Reh cell-free extracts. Cell-free extracts (1.0 *μ*g) were incubated with oligomeric DNA possessing 10 nt microhomology in a buffer containing 10 mM Tris⋅HCl (pH 8.0), 20 mM MgCl_2_, 1 mM ATP, 10% PEG 8000 and 1 mM DTT for 2 h at 30 °C. The products were resolved on a PAGE and visualized. For other details refer Figure 2 legend. M is 50 nt ladder. M is marker for 60 nt position. MMEJ and NHEJ products are indicated. (**d**) Bar diagram showing comparison of MMEJ and C-NHEJ catalyzed by testis, thymus, K562 and Reh, relative to input. *P<0.05. Error bars represent S.E.M. (**e**) Comparison of different modes of NHEJ among normal tissues and cancer cell lines. The total end-joining junctions from testis, thymus, K562 and Reh cells were PCR amplified, cloned and sequenced. Each sequence shown is derived from an independent clone. Cases where microhomology is used are indicated in the column. The ‘joined products' indicate the usage of substrates. Red color indicates sequences that are deleted, while blue indicates insertions. Green indicates mutations in the sequence. Microhomology region is underlined and the sequence is indicated in bold. (**f**) Western blots showing expression profile of canonical and noncanonical NHEJ proteins in testes, thymus and leukemic cell lines. Bands marked with an asterisk could be an isoform. Both isoforms of Ligase III, *α* and *β*, are indicated. PCNA and actin were used as loading control

In order to investigate MMEJ in normal tissues, cell-free extracts were prepared from rat testes and thymus and used for cell-free DSB repair assay. Double-stranded DNA containing microhomology were incubated in extracts (normal tissues and cancer cells, for 2 h at 30 °C; Figures 3a–d). Results showed detectable MMEJ in both normal tissues and cancer cell lines. MMEJ was more prominent in testes compared with thymus among normal tissues (Figures 3a–d). Upon comparing the relative intensities of MMEJ products with input DNA substrates, results indicated that 2–3% of DNA molecules would have joined using microhomology in different cellular extracts (Figure 3d). Besides MMEJ, we could detect joining due to C-NHEJ despite using substrates that promote microhomology-mediated joining (Figures 3c and d). Interestingly, varying levels of MMEJ were observed in other normal tissues such as spleen, lungs, heart, liver and kidney (Supplementary Figure 2). Thus, our study revealed an efficient MMEJ in normal tissues.

Cloning and sequencing of end-joined junctions from independent biological repeats revealed that MMEJ could take place in both normal and cancer cells by utilizing microhomology (Figure 3e,Supplementary Figure 3). End-joined junctions were also due to C-NHEJ, which was characterized by small deletions or insertions (Figure 3e,Supplementary Figure 3). These results confirmed the occurrence of microhomology-mediated joining in normal cells. Western blot analysis showed efficient expression of RAD50, MRE11, ATM, PARP1, LIGASE IV and LIGASE III in all four cell types (Figure 3f). Besides, we observed varying expression of APLF, BRCA1, WRN and LIGASE I in normal and cancer cells (Figure 3f).

### Role of microhomology in alternative end joining

**Figure 4:**
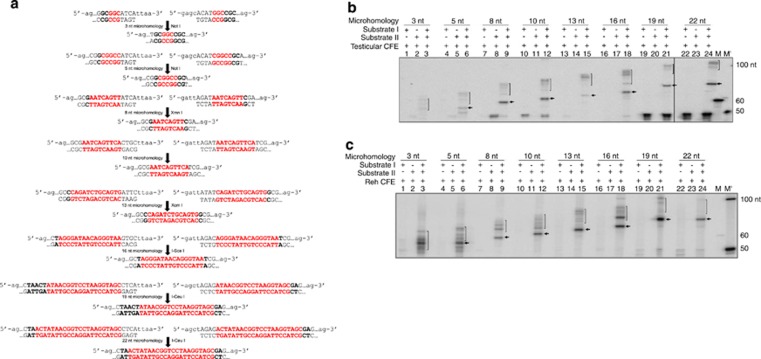
Determination of length of microhomology requirement during MMEJ. (**a**) Depiction of sequence and probable MMEJ product following joining of ds oligomeric substrates possessing 3, 5, 8, 10, 13, 16, 19 and 22 nt of microhomology, which are indicated in red. Restriction enzyme sites generated due to microhomology-mediated joining are also indicated. (**b**) Comparison of MMEJ catalyzed by testicular extracts when different microhomology regions were used. Rat testicular extracts were incubated with oligomeric DNA substrates harboring 3, 5, 8, 10, 13, 16, 19 and 22 nt microhomology for 2 h at 25 ºC. End-joined products were detected by radioactive PCR. In case of every substrate, reactions are shown with either of the substrate or both. MMEJ products are indicated by arrow, while NHEJ products are bracketed. M and M' are molecular weight markers. (**c**) Comparison of MMEJ of oligomeric DNA containing DSBs when flanked with different length microhomology catalyzed by Reh cell-free extract. M and M' are markers as indicated

In order to understand the extent of microhomology required for MMEJ, oligomeric DNA containing DSBs flanked by various lengths of microhomology (3, 5, 8, 10, 13, 16, 19 or 22 nt) were designed and synthesized in such a way that following MMEJ a restriction enzyme site would be generated (Figure 4a and Supplementary Tables 2 and 4A). Results showed a prominent band due to MMEJ in all the substrates, except for 3 nt microhomology, when incubated with testicular extracts (Figure 4b). In order to corroborate this observation, PCR products obtained from 3, 5, 8 and 13 nt microhomology substrates following MMEJ reaction, were digested with NotI, XmnI and XcmI, respectively. A low-intensity band corresponding to ~55 nt in 5 nt microhomology substrate was detected, which was digestible with NotI indicating that the joining was mediated by MMEJ (Supplementary Figure 4b). Similarly, bands corresponding to ~60 and 65 nt in 8 and 13 nt microhomology substrates were digestible with XmnI and XcmI, respectively (Supplementary Figure 4b). Besides, we could not find any specific band, which was sensitive to NotI digestion when 3 nt microhomology substrate was used (Supplementary Figure 4b). As there was no suitable restriction enzyme site, we could not perform similar analysis for substrates containing 10 nt microhomology. These findings suggest that MMEJ protein machinery recognizes microhomology regions to undergo end joining. Furthermore, a microhomology length of more than 3 nt is essential for its operation.

Comparison of MMEJ in thymic cell-free extracts showed an increase in the efficiency of joining with an increase in length of microhomology (data not shown). Similar to testicular extracts, we could not detect any joining due to MMEJ, in case of 3 nt microhomology-containing DNA substrates. MMEJ was observed in K562 and Reh cell-free extracts, when substrate containing as low as 5 nt microhomology was used (Figure 4c and data not shown). Further, a definitive enhancement in the MMEJ efficiency was observed with an increase in length of microhomology, which was consistent with the results seen in budding yeast.^22^ In all cases, reaction products due to joining through NHEJ were also seen (Figure 4c).

**Figure 5:**
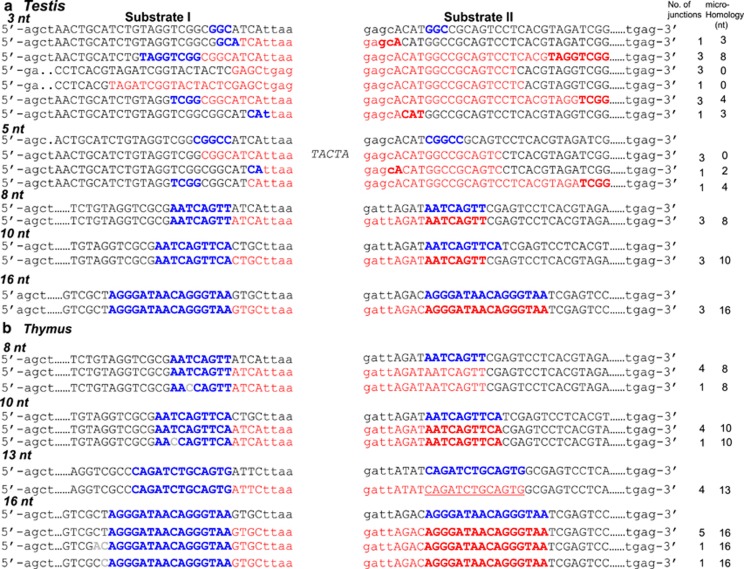
Sequence analysis of MMEJ junctions, when DSBs with different microhomology regions were used. Products due to possible MMEJ (3, 5, 8, 10, 13 and 16 nt microhomology) catalyzed by testicular and thymic extracts were gel purified, cloned and sequenced. The sequences denoted in red color are the deleted nucleotides. (**a**) MMEJ junctions from rat testicular extracts. (**b**) MMEJ junctions derived from rat thymic extracts. For other details, refer Figures 1 and 4 legends

In order to confirm the identity of potential MMEJ products, corresponding bands were excised, purified and subjected to DNA sequencing for 3, 5, 8, 10, 13 and 16 nt microhomology-containing substrates. Results confirmed the presence of MMEJ in case of each substrate, when the microhomology was 8 nt or more (Figure 5a). In case of 5 nt microhomology substrates, only few junctions utilized microhomology (based on restriction digestion analysis), whereas majority of the joining was through C-NHEJ. In case of 3 nt microhomology substrates, none of the clones showed usage of microhomology. Unlike C-NHEJ, MMEJ junctions did not show any insertions, although point mutations were rarely seen. Similar results were also seen when MMEJ junctions were sequenced from thymus (Figure 5b). These results suggest that the MMEJ is dependent on the length of microhomology region.

### MMEJ is not dependent on classical NHEJ proteins

**Figure 6:**
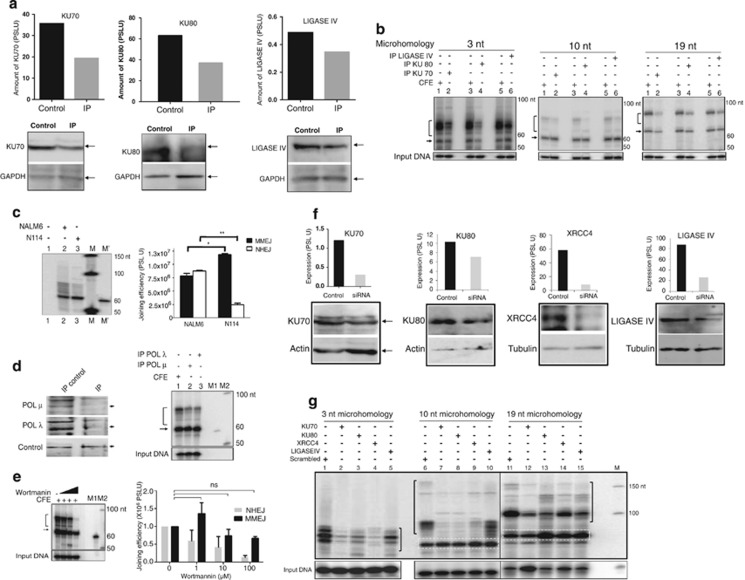
Evaluation of the effect of C-NHEJ proteins on joining of DNA substrates containing DSBs flanked with 3, 10 and 19 nt microhomology regions. (**a**) Western blots showing immunodepletion of KU70, KU80 and LIGASE IV proteins from rat testicular cell extracts. ‘Control' is whole cell extract and ‘IP' is immunodepleted extract. GAPDH was used as an internal loading control. (**b**) Efficiency of MMEJ and NHEJ following immunodepletion of KU70, KU80 and LIGASE IV on DNA substrates containing 3, 10 and 19 nt microhomology. (**c**) Comparison of MMEJ and C-NHEJ in NALM6 and Ligase IV genetic knockout cell lines. Cell-free extracts were incubated with 10 nt microhomology substrates as described, products from multiple experiments were quantified and presented. *P<0.05; **P<0.01. Error bars represent S.E.M. (**d**) Western blots showing immunodepletion of polymerase *μ* and *λ* and evaluation of MMEJ efficiency using the immunodepleted extracts. (**e**) Effect of wortmannin, a DNA-PKcs inhibitor on MMEJ. Wortmanin (1, 10 and 100 *μ*M) was incubated with cell-free extracts of testis (2 *μ*g) and oligomeric DNA containing 10 nt microhomology and analyzed. In panels **b**–**e**, MMEJ products are indicated by arrow, while NHEJ products are bracketed. (**f**) siRNA-mediated knockdown of classical NHEJ proteins in Reh cells. Reh cells were transfected with siRNA against KU70, KU80, XRCC4 and LIGASE IV and harvested after 48 h. Cell-free extracts were prepared and efficiency of knockdown was evaluated by western blotting and was quantified (shown as bar diagram). Scrambled siRNA was used as control. (**g**) Efficiency of MMEJ and NHEJ following siRNA-mediated knockdown of expression of KU70, KU80, XRCC4 and LIGASE IV. MMEJ products are boxed, while NHEJ products are bracketed. M is marker

As we could distinguish MMEJ products using cell-free repair system, we were interested in protein machinery responsible for alternative NHEJ. To begin with, possible involvement of C-NHEJ proteins like KU70, KU80 and LIGASE IV was tested by immunoprecipitation (IP) (Figure 6a) followed by joining assay. No effect on MMEJ products (10 or 19 nt microhomology) was observed when immunodepleted extracts of KU70, KU80 and LIGASE IV were used (Figure 6b). However, a significant decrease in the efficiency of C-NHEJ products was observed. Further, we could find an overall decrease in the efficiency of joining when 3 nt microhomology-containing substrate was used (Figure 6b). Consistent with these findings, when cell-free extracts were prepared from LIGASE IV knockout cells, there was no reduction in MMEJ efficiency, whereas C-NHEJ was significantly inhibited (Figure 6c). Besides, immunodepletion of POL *μ* or POL *λ* also did not result in any significant reduction in the efficiency of MMEJ, whereas levels of NHEJ products were substantially reduced (Figure 6d).

Wortmannin, an inhibitor of DNA-PKcs, is known to block NHEJ in its initial stages.^25^ Testicular extracts were incubated with increasing concentrations of wortmannin (1, 10 and 100 *μ*M) and tested for its effect on MMEJ by using 10 and 19 nt microhomology substrates (Figure 6e,Supplementary Figure 5). Results showed a reduction in NHEJ products upon an increase in concentrations of wortmannin in case of 10 nt microhomology substrates, whereas the efficiency of MMEJ remained unaltered (Figure 6e). However, at highest concentration of wortmannin, a minor reduction in MMEJ was observed, which could be attributed to the ability of wortmannin to inhibit other PI 3-kinases (Figure 6e).^26^ Inhibition of C-NHEJ along with an increase in MMEJ could be observed with 19 nt microhomology substrate upon treatment with wortmannin (Supplementary Figure 5). Thus, this result, in conjunction with above findings, suggests that MMEJ is not dependent on classical NHEJ proteins.

In order to test whether the results obtained using the cell-free repair system are relevant at the cellular level, siRNA-mediated knockdown of C-NHEJ proteins was performed. siRNA against KU70, KU80, XRCC4 or LIGASE IV were transfected in Reh cells and cell-free extracts were prepared along with scrambled siRNA control. Knockdown of respective protein was confirmed by western blotting (Figure 6f). When these cell-free extracts were used for MMEJ assay on substrates with DSBs flanked with 3, 10 and 19 nt microhomology, an overall reduction in the efficiency of joining of 3 nt microhomology substrate was observed (Figure 6g). In contrast, substrates possessing 10 and 19 nt microhomology, showed improved efficiency of MMEJ upon knockdown of KU70, KU80, XRCC4 or LIGASE IV (Figure 6g). As expected, C-NHEJ products exhibited a remarkably low efficiency of joining (Figure 6g). Hence, siRNA knockdown within the cells in conjunction with immunodepletion studies suggest that C-NHEJ proteins are not involved during MMEJ.

### Microhomology-mediated alternative NHEJ is dependent on other repair proteins

**Figure 7:**
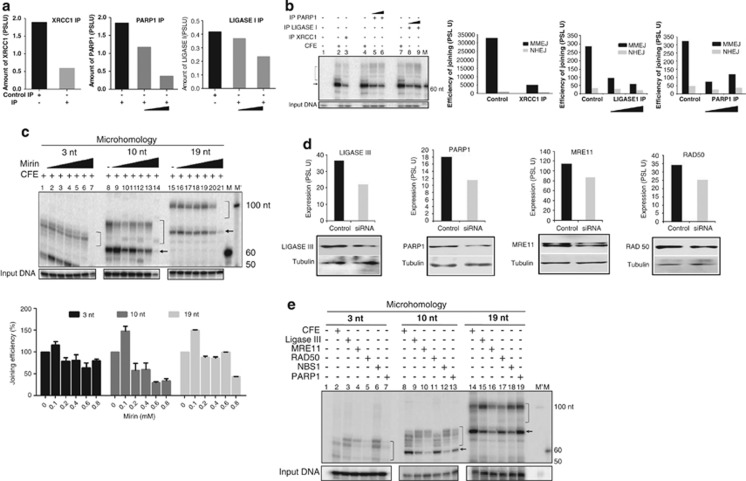
Determination of proteins responsible for microhomology-mediated alternative end joining. (**a**) Bar diagram showing immunodepletion of XRCC1, PARP1 and LIGASE I proteins from rat testicular extracts. Lane 1, control IP. Lanes 2 and 3 are immunodepleted extract following incubation with two different concentrations of antibody (0.2 and 0.3 *μ*g/50 *μ*l). Following quantitation data is normalized against respective loading controls and presented. (**b**) Efficiency of MMEJ and C-NHEJ following immunodepletion of XRCC1 and PARP1 and LIGASE I on DNA substrates containing 10 nt microhomology. Joining assay and its quantification (%) are shown. The highest concentration (1 mM) was not considered for quantification, as it inhibited the joining in a nonspecific manner. (**c**) Effect of increasing concentrations of mirin, a MRN complex inhibitor, on MMEJ. Mirin (100, 200, 400, 600, 800 *μ*M and 1 mM) was incubated with cell-free extracts of testis and 3, 10 and 19 nt microhomology-containing substrates and analyzed. Joining assay and its quantitation are presented. (**d**) siRNA-mediated knockdown of proteins in Reh cells. Reh was transfected with siRNA against LIGASE III, PARP1, MRE11 and RAD50, and harvested after 48 h. Cell-free extracts were prepared and efficiency of knockdown was evaluated by western blotting and quantified (shown as bar diagram). Scrambled siRNA was used as control. (**e**) Efficiency of MMEJ and NHEJ following siRNA-mediated knockdown of expression of LIGASE III, PARP1, MRE11, NBS1 and RAD50. MMEJ products are indicated by arrow, while NHEJ products are bracketed. M and M' are markers

As MMEJ is independent of C-NHEJ proteins, we were interested in identifying the proteins involved in MMEJ. On the basis of previous studies, we selected candidate protein targets for the study. Immunodepletion of XRCC1, PARP1 and LIGASE I was performed from rat testicular extracts (Figure 7a) and used for MMEJ on a 10-nt microhomology substrate. Interestingly, results showed a significant decrease in the efficiency of MMEJ, whereas C-NHEJ remained same in case of XRCC1-, LIGASE I- and PARP1-immunodepleted extracts as compared with that of control (Figure 7b). This suggests that MMEJ utilizes protein machinery consisting of XRCC1, LIGASE I and PARP1.

Mirin inhibits MRE11, an enzyme involved in homologous recombination and suggested to have a potential role in A-NHEJ.^27^ Testicular extracts were incubated with increasing concentrations of mirin (100, 200, 400, 600, 800 and 1000 *μ*M) and used for MMEJ reaction with 3, 10 and 19 nt microhomology substrates. Results showed reduction in the formation of MMEJ products with increasing concentrations of mirin, whereas its effect on NHEJ was minimal (Figure 7c), except at higher concentrations, which could be attributed to nonspecificity. These results indicate the possible involvement of MRN complex in MMEJ.

siRNA-mediated knockdown was performed in Reh cells to evaluate the role of MRE11, RAD50, NBS1, PARP1, FEN1, ARTEMIS and LIGASE III in MMEJ in physiological context. Cell-free extracts were prepared from experimental and scrambled siRNA-treated cells. Following confirmation of the knockdown by western blotting (Figure 7d), MMEJ assay was carried out on substrates harboring 3, 10 or 19 nt microhomology. Results showed a distinct reduction in MMEJ when MRE11, NBS1, LIGASE III, PARP1 expressions were knocked down in case of substrates possessing 10 and 19 nt microhomology (Figure 7e). Interestingly, inhibition of RAD50 expression did not result in significant reduction in MMEJ in case of 10 nt microhomology-containing substrate (Figure 7e). However, we did not observe any significant difference in the efficiency of C-NHEJ products upon knockdown of these genes (Figure 7e). Consistent with previous results, there was no significant reduction in the efficiency of joining of 3 nt microhomology substrate (Figure 7e). Results showed a reduction in MMEJ, when FEN1 was knocked down, whereas ARTEMIS knockdown did not show any significant reduction in MMEJ (Supplementary Figure 6). As expected, ARTEMIS knockdown resulted in lower C-NHEJ, whereas FEN1 did not affect its efficiency (Supplementary Figure 6). Hence, our results suggest that MRE11, NBS1, PARP1, XRCC1, FEN1, LIGASE I and LIGASE III are essential for microhomology-mediated joining.

## Discussion

DSBs generated either physiologically or pathologically need to be repaired to maintain the integrity of genome in order to ensure the survival of a cell.^3^ Multiple NHEJ pathways have been proposed for repairing various types of DSBs. Recently, a DNA-PK- and/or KU-independent alternative pathway has been suggested to repair DSBs using microhomology, and to be responsible for deletions and chromosomal translocations.^4, 28, 29^ Microhomology-mediated joining is a major form of alternative NHEJ and has been suggested to operate in cells that are deficient in C-NHEJ.^6^ MMEJ is utilized when C-NHEJ is compromised; however, the extent of its activity in normal physiological conditions is a question yet to be resolved.^23, 28, 30^

### MMEJ is dependent on microhomology and occurs in normal cells

In the present study, we have established a biochemical system to study MMEJ. By using such a system, we are able to detect MMEJ even in normal cells, when microhomology regions are provided. More importantly, this assay system helps in distinguishing joining mediated by both MMEJ and C-NHEJ simultaneously. This suggests that although classical NHEJ is normally predominant, MMEJ can occur in both normal and cancer cells, when DSBs flank the microhomology regions.

Previously, we have shown that efficiency and mode of DSB joining is dependent on DNA end configurations.^31^ In the present study, when DNA possessing different microhomology (3–22 nt) were used, a length of 3 nt microhomology was insufficient to recruit MMEJ machinery; instead C-NHEJ was preferred. Importantly, a 5-nt microhomology was sufficient to elicit MMEJ consistent with the earlier results.^22^ However, MMEJ efficiency was improved when longer microhomology was present. Class switch recombination occurs at 20–50% of the stimulated cells and several joining junctions use longer microhomology in C-NHEJ-deficient B cells.^8^ Preferential joining using 7 nt microhomology was reported in KU80- and XRCC4-deficient cells.^32^ Therefore, length of microhomology is one of the factors that determine the recruitment of the protein machinery for MMEJ. Previously, plasmid-based biochemical assay systems were used to study backup NHEJ pathways using cell lines deficient for DSB repair proteins.^33, 34, 35^

### MMEJ utilizes specific protein machinery and can coexist along with C-NHEJ

Our results showed that in physiological scenario, the set of proteins required for C-NHEJ and MMEJ are distinct. Specifically, during MMEJ, following the end resection, microhomology regions are aligned in such a way that intact product formation takes place following processing of the flap region and ligation. On the basis of our studies, it appears that the efficiency of joining by MMEJ is particularly more favorable when the substrates possess ends with microhomology, hence exhibiting lower efficiency by C-NHEJ.^31^ Besides, based on the studies from our group and others, it appears that there is a complete set of proteins that can perform MMEJ.^5, 6, 8, 9, 11, 18, 19, 36^ In the physiological context, when some of the ends are left unrepaired, it is possible that this repair machinery takes over the process to repair such DNA ends. However, other than microhomology, the additional factors contributing toward the selection of such alternative pathway, within cells, are yet to be understood. Mutation in one of the C-NHEJ proteins alone may not be solely responsible. Recently, MMEJ has been described as the repair mechanism involved in generation of chromosomal translocations.^8^ The fact that many such rearrangements are reported in remarkably higher frequency in healthy individuals further confirm that alternative pathway of NHEJ might be operating in normal scenario as well.^37, 38, 39, 40, 41, 42^ It is well known that the first hit of genetic alterations like chromosomal translocations and deletions, which lead to carcinogenesis, occur in noncancerous cells. Thus, our observations of MMEJ in normal cells are of great importance.

### Microhomology-mediated alternative NHEJ is dependent on MRE11, NBS1, PARP1 and LIGASE III*α*/XRCC1 complex

**Figure 8:**
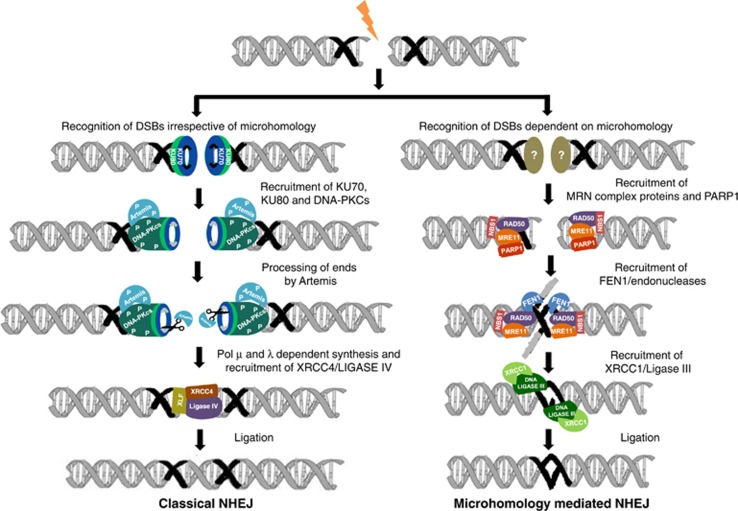
Mechanism of microhomology-mediated alternative NHEJ. Schematic presentation shows different modes of DSB repair via NHEJ and MMEJ. When a DSB is generated flanking a microhomology region, there are two outcomes. If KU proteins bind to the ends and recruit C-NHEJ proteins, it can result in C-NHEJ. Alternatively, if the microhomology is recognized by MRN complex and PARP1 or a yet unidentified protein, it can follow MMEJ. This is followed by recruitment of FEN1/unknown endonucleases, which can remove the flap. Further, recruitment of XRCC1–LIGASE III at the site helps in ligating the DNA ends leading to an intact DNA

By using both immunodepletion along with inhibitor studies in cell-free extracts and siRNA-mediated knockdown within the cells, we deciphered that classical NHEJ proteins are not required for microhomology-mediated alternative NHEJ (Figure 8). Instead, we found that proteins such as PARP1, XRCC1, LIGASE III, MRE11, FEN1 and NBS1 are important for MMEJ (Figure 8). As MRN complex has an end resection property, it searches for homology and once aligned, DSBs are joined using LIGASE III/XRCC1.^43^ Alternatively, it is possible that a yet unidentified protein recognizes the microhomology region and recruits MRN complex or PARP proteins to the site of repair. Role of MRE11 in alternative NHEJ has been shown earlier.^9^ Here we observed a significant reduction in the intensity of MMEJ product and a slight reduction in C-NHEJ products upon removal of MRN complex in case of substrates containing either 10 or 19 nt microhomology. Earlier studies have shown that depletion or inhibition of MRE11 reduces end joining in wild-type and XRCC4^−/−^ cells showing that MRE11 promotes both C-NHEJ and MMEJ.^13, 14^ Studies have also shown the potential role of PARP1 and DNA LIGASE III in alternative pathway of NHEJ using *in vivo* plasmid rejoining assay.^34^ DNA LIGASE III as a candidate component of backup end joining was suggested by fractionation studies using plasmid rejoining assay.^35^ Further, it has been shown that involvement of XRCC1–LIGASE III was independent of DNA-PK and XRCC4–LIGASE IV.^44^

Hence, our study establishes a well-defined biochemical system for the characterization of microhomology-mediated alternative NHEJ. It opens a new window to understand the relative frequency of classical NHEJ and MMEJ under physiological conditions. It might also help to interpret the physiological conditions where one pathway may be chosen over the other while repairing DNA DSBs, which could further help in understanding the generation of chromosomal translocations leading to cancer.

## Materials and Methods

### Enzymes, chemicals and reagents

Chemical reagents were obtained from Sigma Chemical Co. (St Louis, MO, USA), Amresco (Solon, OH, USA), SRL (Mumbai, India) and Himedia (Mumbai, India). DNA-modifying enzymes were from New England Biolabs (Beverly, MA, USA) and antibodies were purchased from Santa Cruz Biotechnology (Dallas, TX, USA). Radioisotope-labelled nucleotides were purchased from BRIT (Hyderabad, India).

### Preparation of DNA substrates for MMEJ

Oligomers containing appropriate microhomology regions were designed and commercially synthesized (Supplementary Table 1). The oligomers were then purified on denaturing polyacrylamide gels as described.^45^ In order to generate double-stranded oligomeric DNA containing DSBs with flanking microhomology regions, complementary oligomers were annealed in 100 mM NaCl and 1 mM EDTA, as described.^31^ Length of microhomology region used in the study is 3, 5, 8, 10, 13, 16, 19 and 22 nt (Supplementary Table 2). The 5′ end-labelling ((*γ*-^32^P) ATP) of the SS60 and other oligomers was done using T4 polynucleotide kinase as described previously^46^ and stored at −20 ^o^C.

### Preparation of cell-free extracts from tissues and cell lines

Cell-free extracts were prepared from testes and thymus of male winstar rats (3–4 weeks), as described earlier.^31, 47^ Extracts were aliquoted and stored at −80 °C till use. Protein concentration was determined by Bradford's assay. Protein amount was normalized further by loading on SDS polyacrylamide gel, followed by staining with Coomassie Brilliant Blue.

Cell-free extracts from cell lines K562 (human immortalized myelogenous leukemia line) and Reh (acute lymphocytic leukemia line) were prepared as described.^25^ Briefly, cells were washed with PBS followed by washing in hypotonic lysis buffer (10 mM Tris⋅HCl (pH 8.0), 1 mM EDTA and 5 mM DTT). Cells were resuspended in 100 *μ*l of hypotonic buffer followed by homogenization after 20 min, and protease inhibitors were added (phenylmethylsulfonyl fluoride, 0.01 M; aprotinin, 2 *μ*g/ml; pepstatin, 1 *μ*g/ml; leupeptin, 1 *μ*g/ml). After 20 min on ice, 50 *μ*l of high-salt buffer (50 mM Tris⋅HCl (pH 7.5), 1 M KCl, 2 mM EDTA and 2 mM DTT) was added, and the extract was centrifuged for 3 h at 42 000 r.p.m. at 4 °C in a Beckman TLA-100 Rotor (Beckman, Palo Alto, CA, USA). The supernatant was dialyzed overnight against dialysis buffer (20 mM Tris⋅HCl (pH 8.0), 0.1 M KOAc, 20% glycerol, 0.5 mM EDTA and 1 mM DTT), snap frozen and stored at −80 °C.

### Evaluation of MMEJ

DNA end joining assay was performed by incubating 4 nM of radiolabelled oligomeric DNA containing microhomology in cell-free extracts (1 *μ*g), unless specified otherwise (testicular cells) in a buffer (10 mM Tris⋅HCl (pH 8.0), 20 mM MgCl_2_, 1 mM ATP, 10% PEG 8000 and 1 mM DTT) in a reaction volume of 20 *μ*l at 30 °C for 2 h or as specified (Figure 1a). End-joining reactions were arrested by heat inactivation of proteins at 65 °C for 20 min. Products were detected by PCR using (*γ*-^32^P) ATP-labelled primer, SS60 and unlabelled primer SS61 (denaturation: 95 °C for 3 min (1 cycle); denaturation: 95 °C for 30 s, annealing: 58 °C for 45 s, extension: 72 °C for 30 s (15 cycles); extension: 72 °C for 3 min (1 cycle)). Reaction products were resolved on an 8% denaturing polyacrylamide gel, and signals were acquired using a PhosphorImager (GE, Pittsburgh, PA, USA). The quantity of input DNA in the PCR reaction was determined by the amplification of the longer MMEJ substrates using primers (MS143 and MS144), which served as the control. Each experiment described in the present study was performed a minimum of three independent times. Results were in good agreement and representative gels with quantifications are shown.

### Cloning and sequencing of end-joined junctions

For cloning of total reaction products, PCR was performed using primers SS60 and SS61. For cloning of prospective MMEJ products, the band of interest was excised and purified from the gel and used for PCR amplification using same set of primers. In all cases, PCR products were purified and cloned into TA vector as described.^48^ The presence of insert was confirmed by restriction enzyme digestion followed by DNA sequencing (SciGenom Labs Pvt Ltd., Cochin, India).

### siRNA transfection

Reh cells were used for siRNA transfection studies. Briefly, Reh cells (2.5 × 10^6^) were transfected with a cocktail of siRNA against KU70, KU80, XRCC4, LIGASE IV, MRE11, RAD50, NBS1, PARP1 or LIGASE III using oligofectamine as described earlier.^49^ The cells were harvested after 48 h and cell-free extracts were prepared as described above. The efficiency of knockdown was confirmed using immunoblotting and used for assessing DNA end joining.

### Immunoblotting and IP

For immunoblotting analysis, 20–40 *μ*g of protein was resolved on 7–10% SDS polyacrylamide gel.^49, 50^ Following electrophoresis, proteins were transferred to PVDF membrane (Millipore, Billerica, MA, USA), and probed with appropriate primary antibodies against KU70, KU80, XRCC4, LIGASE IV, POL *λ*, POL *μ*, MRE11, WRN, CtIP, APLF, PARP1, BRCA1, RAD50, NBS1, LIGASE I and LIGASE III (Santa Cruz Biotechnology) and appropriate secondary antibodies as per standard protocol. TUBULIN, GAPDH, PCNA or ACTIN were used as internal loading controls. The blots were developed using chemiluminescent solution (Immobilon Western, Millipore) and scanned by gel documentation system (LAS 3000, Fuji, Shizuoka, Japan).

IP experiments were performed independently for respective proteins as described previously.^31, 51^ Protein G agarose beads (Sigma, St. Louis, MO, USA) were incubated with appropriate antibody (0.04 *μ*g/*μ*l) overnight in IPP buffer (0.5 M NaCl, 10 mM Tris (pH 7.5) and 0.2% NP-40). The beads were spun down and the supernatant was removed. A unit of 50 *μ*g of rat testicular extract was then mixed with the antibody-bound beads and incubated. The immunodepletion was confirmed by western blot analysis. Images were quantified using Multi Gauge (V3.0; Fujifilm, Tokyo, Japan) and presented as bar diagram. The immunodepleted extract was then used for MMEJ assay.

### Quantification

For quantification of bands of interest, Multi Gauge (V3.0) software was used as described.^31^ A rectangle was selected covering the band of interest and the intensity was quantified. A similar rectangle was then placed over other bands of interest in each lane, quantified and added. An equal area from the same lane of the blot where no specific band was present was used as background and subtracted. The intensity obtained from each lane was plotted and presented as bar diagram.

### Statistical analyses

Values are expressed as mean±S.E.M. for control and experimental samples, and statistical analyses were performed by one-way ANOVA followed by unpaired Student's *t*-test with GraphPad Prism 6 software (San Diego, CA, USA). The values were considered statistically significant, if the *P*≤0.05.

### DNA end-joining assay in presence of MRE11 and DNA-PKcs inhibitors

Reaction with wortmannin (DNA-PKcs inhibitor) or mirin (MRE11 inhibitor) was carried out as described.^25^ Different concentrations of inhibitors were incubated with protein extracts for 30 min on ice, shifted to 25 °C for 15 min, and then 4 nM of substrate was added and incubated for 2 h. End-joined junctions were then PCR amplified and loaded onto 8% denaturing polyacrylamide gel. The images were acquired as described above.^52, 53^

## Abbreviations

CSR: class switch recombination
NHEJ: nonhomologous DNA end joining
DSB: double-strand break
MMEJ: microhomology-mediated end joining
C-NHEJ: classical nonhomologous DNA end joining
A-NHEJ: alternative nonhomologous DNA end joining
ATP: adenosine triphosphate
IP: immunoprecipitation
