# Supplementary material for: Homology and enzymatic requirements of microhomology-dependent alternative end joining
Source: Cell Death Dis. 2015 Mar 19;6(3):e1697–. doi: 10.1038/cddis.2015.58 (PMC4385936; doi:10.1038/cddis.2015.58)
Supplement: Supplementary Figures Legends [file cddis201558x1.doc]

**SUPPLEMENTARY MATERIALS**

**Supplementary Figure Legends**

**Supplementary Table 1.** List of oligomers used in the study.

**Supplementary Table 2.** List oligomers used for preparing DNA substrates containing different lengths of microhomology (3, 5, 8, 10, 13, 16, 19 and 22 nt).

**Supplementary Figure 1. a.** Sensitivity of the Alt-NHEJ assay system as determined by performing reaction with serially diluted DNA substrates (0.002, 0.01, 0.02, 0.1, 0.2, 2, 4 and 8 nM). **b.** Line curve depicting correlation of substrate concentrations (nM) with efficiency of product conversion (PSL units).

**Supplementary Figure 2.** Sequence analysis showing comparison of different modes of NHEJ among normal tissues and cancer cell lines. This is a magnified version of Figure 3e. The total end joining junctions from testis, thymus, K562 and Reh cells were PCR amplified, cloned and sequenced. Each sequence shown is derived from an independent clone. Cases where microhomology is used are indicated. Red color indicates sequences that are deleted, while blue indicates insertions. Green indicates mutations in the sequence. Microhomology region is underlined and the sequence is indicated in bold.

**Supplementary Figure 3. Comparison of MMEJ in normal tissues.** **a.** Cell-free extracts prepared from rat brain, testis, thymus, spleen, lungs, heart, liver and kidney were incubated with 8 nt microhomology substrates followed by radioactive PCR. M and M’ indicates markers.

**Supplementary Figure 4. Evaluation of requirement of microhomology during MMEJ**. **a**. Depiction of sequence and probable MMEJ product following joining of ds oligomeric substrates possessing 3, 5, 8, 10, 13, 16, 19 and 22 nt of microhomology, which are indicated in red. This is a magnified version of Figure 4A. Restriction enzyme sites generated due to microhomology mediated joining are also indicated.  **b.** Restriction digestion analysis of MMEJ products derived from selected microhomology substrates. MMEJ assay was performed with rat testicular extracts followed by purification of products and restriction digestion analysis. The restriction enzyme used and microhomology region analysed are NotI for 3 and 5 bp, XmnI for 8 bp, XcmI for 13 bp. Bands due to MMEJ are indicated by dark arrows and NHEJ products are bracketed. New bands resulting due to restriction digestion are indicated by arrows.

**Supplementary Figure 5. Evaluation of role of DNA-PKcs in MMEJ**. **a.** Increasing concentrations of wortmannin (**0,** 1, 10 and 100 µM) were incubated with rat testicular extracts followed by addition of DNA substrate possessing 19 nt microhomology. MMEJ and C-NHEJ products were detected by radioactive PCR. **b.** Bar diagram representing quantification of MMEJ and C-NHEJ after inhibition of DNA-PKcs in testicular extracts.

**Supplementary Figure 6. Determination of role of nucleases in MMEJ.** siRNA against Artemis and FEN1 were transfected in Reh cells followed and used for the study. Scrambled siRNA was used as control. **a.** Western blotting analysis to evaluate the efficiency of knockdown of Artemis and FEN1 which is quantified and shown as bar diagram. Tubulin was used as loading control. **b**. MMEJ assay to evaluate the efficiency of C-NHEJ and microhomology mediated end joining after knockdown of Artemis and FEN1. For other details refer Figure 6 legend.
