# Supplementary material for: Homology and enzymatic requirements of microhomology-dependent alternative end joining
Source: Cell Death Dis. 2015 Mar 19;6(3):e1697–. doi: 10.1038/cddis.2015.58 (PMC4385936; doi:10.1038/cddis.2015.58)
Supplement: Supplementary Figures and tables [file cddis201558x2.ppt]

## Slide 1
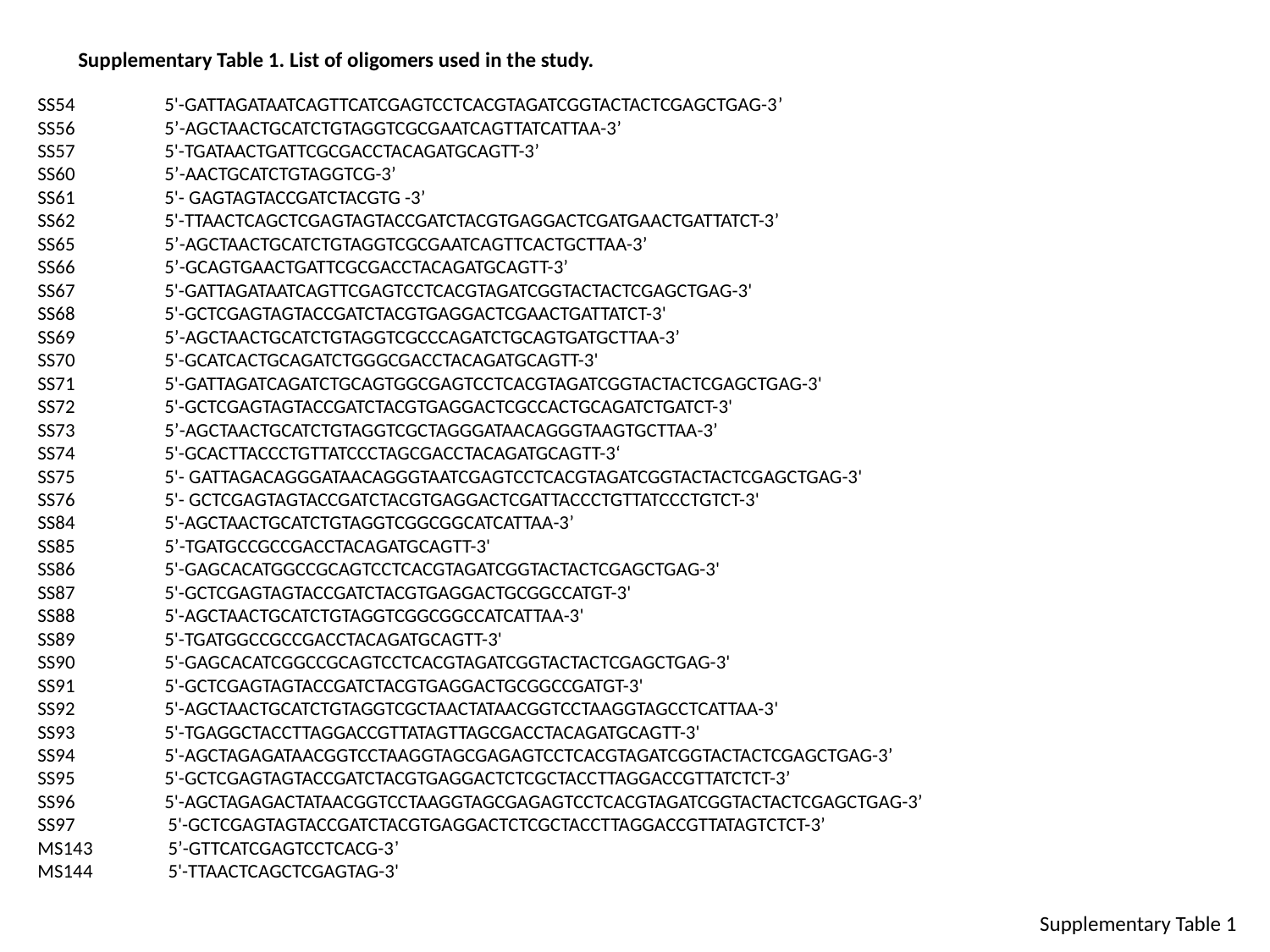

Supplementary Table 1. List of oligomers used in the study.
SS54	5'-GATTAGATAATCAGTTCATCGAGTCCTCACGTAGATCGGTACTACTCGAGCTGAG-3’
SS56	5’-AGCTAACTGCATCTGTAGGTCGCGAATCAGTTATCATTAA-3’
SS57	5'-TGATAACTGATTCGCGACCTACAGATGCAGTT-3’
SS60	5’-AACTGCATCTGTAGGTCG-3’
SS61	5'- GAGTAGTACCGATCTACGTG -3’
SS62	5'-TTAACTCAGCTCGAGTAGTACCGATCTACGTGAGGACTCGATGAACTGATTATCT-3’
SS65	5’-AGCTAACTGCATCTGTAGGTCGCGAATCAGTTCACTGCTTAA-3’
SS66	5’-GCAGTGAACTGATTCGCGACCTACAGATGCAGTT-3’
SS67	5'-GATTAGATAATCAGTTCGAGTCCTCACGTAGATCGGTACTACTCGAGCTGAG-3'
SS68	5'-GCTCGAGTAGTACCGATCTACGTGAGGACTCGAACTGATTATCT-3'
SS69	5’-AGCTAACTGCATCTGTAGGTCGCCCAGATCTGCAGTGATGCTTAA-3’
SS70	5'-GCATCACTGCAGATCTGGGCGACCTACAGATGCAGTT-3'
SS71	5'-GATTAGATCAGATCTGCAGTGGCGAGTCCTCACGTAGATCGGTACTACTCGAGCTGAG-3'
SS72	5'-GCTCGAGTAGTACCGATCTACGTGAGGACTCGCCACTGCAGATCTGATCT-3'
SS73	5’-AGCTAACTGCATCTGTAGGTCGCTAGGGATAACAGGGTAAGTGCTTAA-3’
SS74	5'-GCACTTACCCTGTTATCCCTAGCGACCTACAGATGCAGTT-3‘
SS75	5'- GATTAGACAGGGATAACAGGGTAATCGAGTCCTCACGTAGATCGGTACTACTCGAGCTGAG-3'
SS76	5'- GCTCGAGTAGTACCGATCTACGTGAGGACTCGATTACCCTGTTATCCCTGTCT-3'
SS84	5'-AGCTAACTGCATCTGTAGGTCGGCGGCATCATTAA-3’
SS85	5’-TGATGCCGCCGACCTACAGATGCAGTT-3'
SS86	5'-GAGCACATGGCCGCAGTCCTCACGTAGATCGGTACTACTCGAGCTGAG-3'
SS87	5'-GCTCGAGTAGTACCGATCTACGTGAGGACTGCGGCCATGT-3'
SS88	5'-AGCTAACTGCATCTGTAGGTCGGCGGCCATCATTAA-3'
SS89	5'-TGATGGCCGCCGACCTACAGATGCAGTT-3'
SS90	5'-GAGCACATCGGCCGCAGTCCTCACGTAGATCGGTACTACTCGAGCTGAG-3'
SS91	5'-GCTCGAGTAGTACCGATCTACGTGAGGACTGCGGCCGATGT-3'
SS92	5'-AGCTAACTGCATCTGTAGGTCGCTAACTATAACGGTCCTAAGGTAGCCTCATTAA-3'
SS93	5'-TGAGGCTACCTTAGGACCGTTATAGTTAGCGACCTACAGATGCAGTT-3'
SS94	5'-AGCTAGAGATAACGGTCCTAAGGTAGCGAGAGTCCTCACGTAGATCGGTACTACTCGAGCTGAG-3’
SS95	5'-GCTCGAGTAGTACCGATCTACGTGAGGACTCTCGCTACCTTAGGACCGTTATCTCT-3’
SS96	5'-AGCTAGAGACTATAACGGTCCTAAGGTAGCGAGAGTCCTCACGTAGATCGGTACTACTCGAGCTGAG-3’
SS97 5'-GCTCGAGTAGTACCGATCTACGTGAGGACTCTCGCTACCTTAGGACCGTTATAGTCTCT-3’
MS143 5’-GTTCATCGAGTCCTCACG-3’
MS144 5'-TTAACTCAGCTCGAGTAG-3'
Supplementary Table 1

## Slide 2
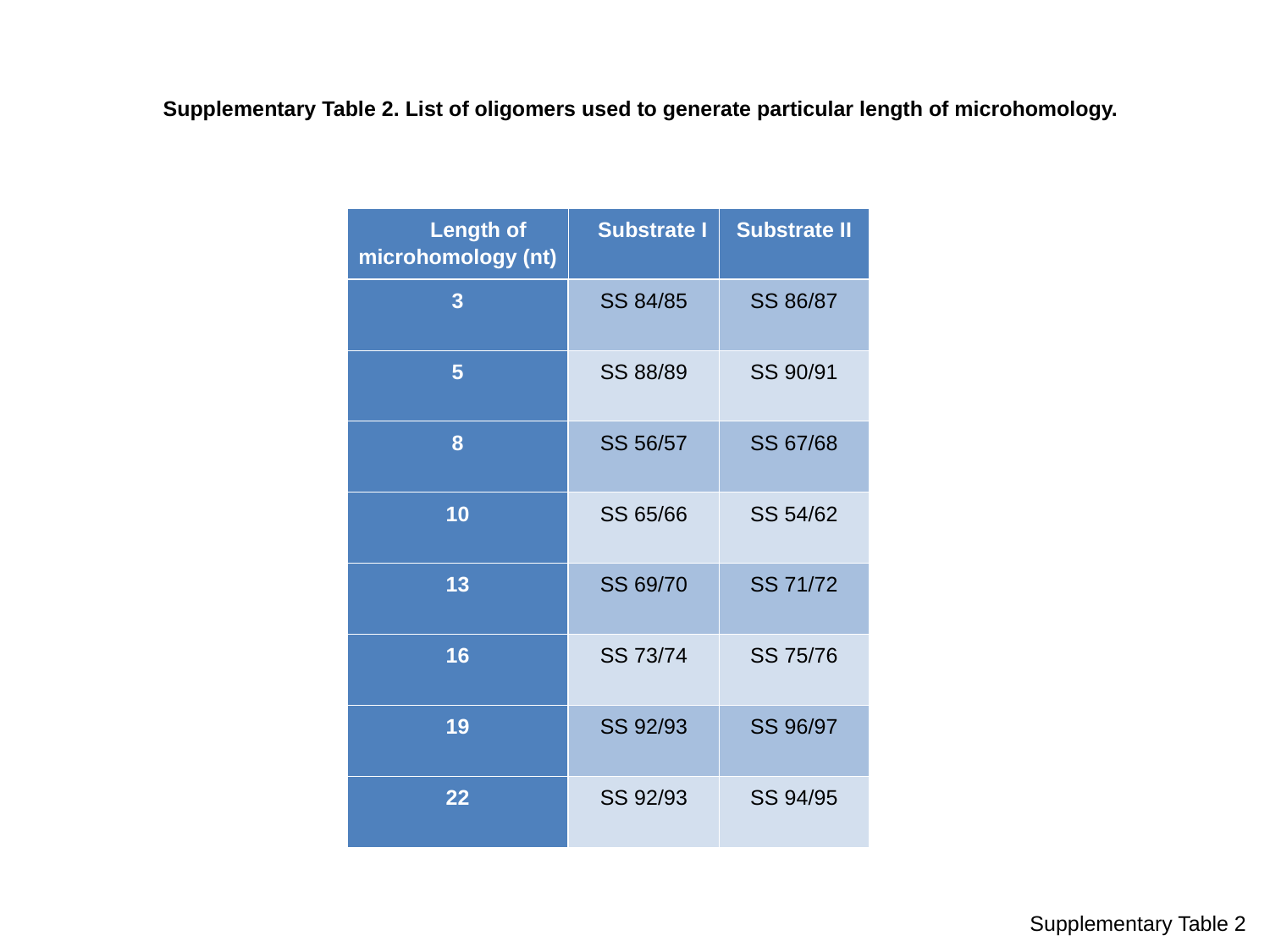

Supplementary Table 2. List of oligomers used to generate particular length of microhomology.
| Length of microhomology (nt) | Substrate I | Substrate II |
| --- | --- | --- |
| 3 | SS 84/85 | SS 86/87 |
| 5 | SS 88/89 | SS 90/91 |
| 8 | SS 56/57 | SS 67/68 |
| 10 | SS 65/66 | SS 54/62 |
| 13 | SS 69/70 | SS 71/72 |
| 16 | SS 73/74 | SS 75/76 |
| 19 | SS 92/93 | SS 96/97 |
| 22 | SS 92/93 | SS 94/95 |
Supplementary Table 2

## Slide 3
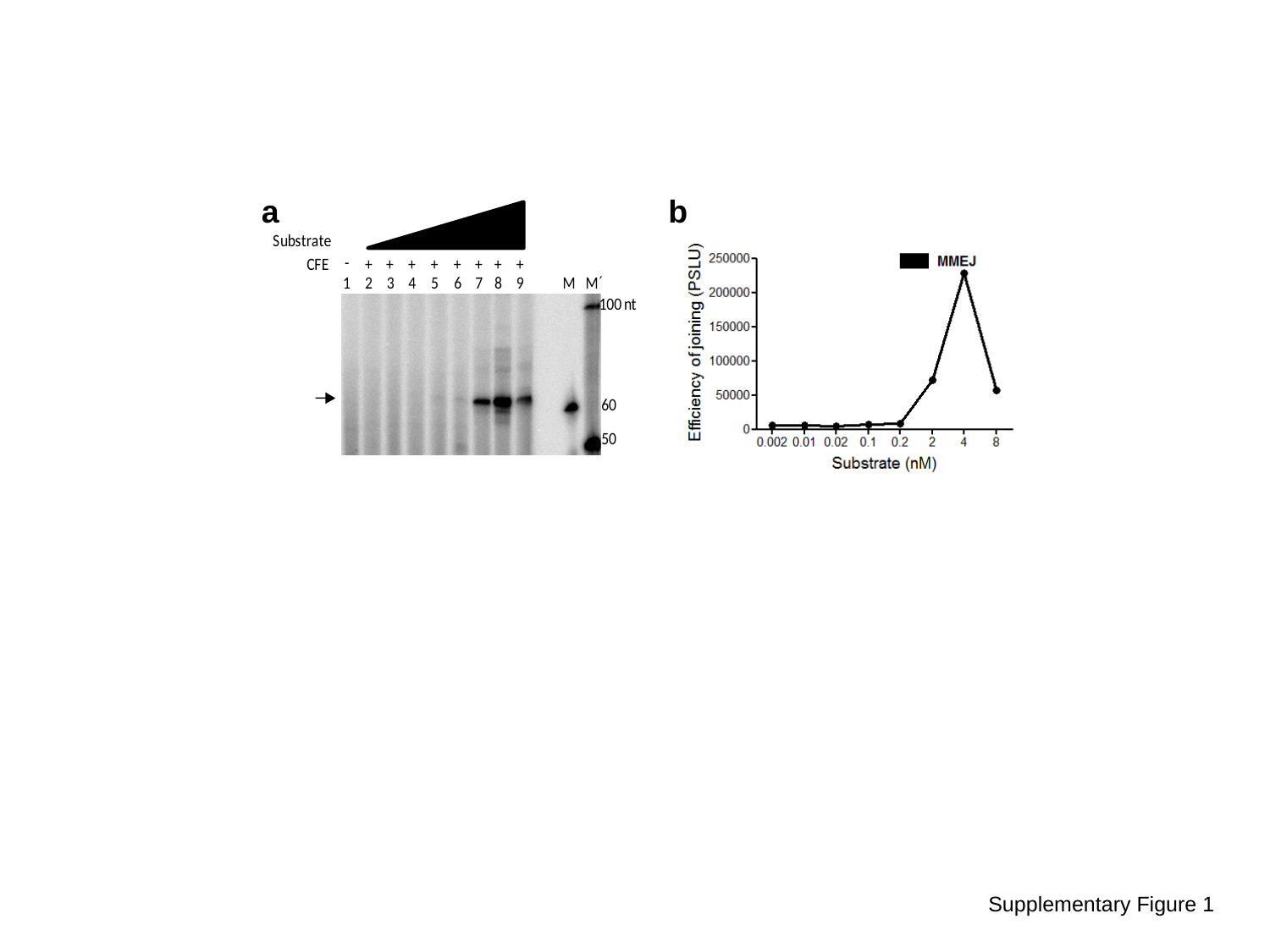

a
b
Supplementary Figure 1

## Slide 4
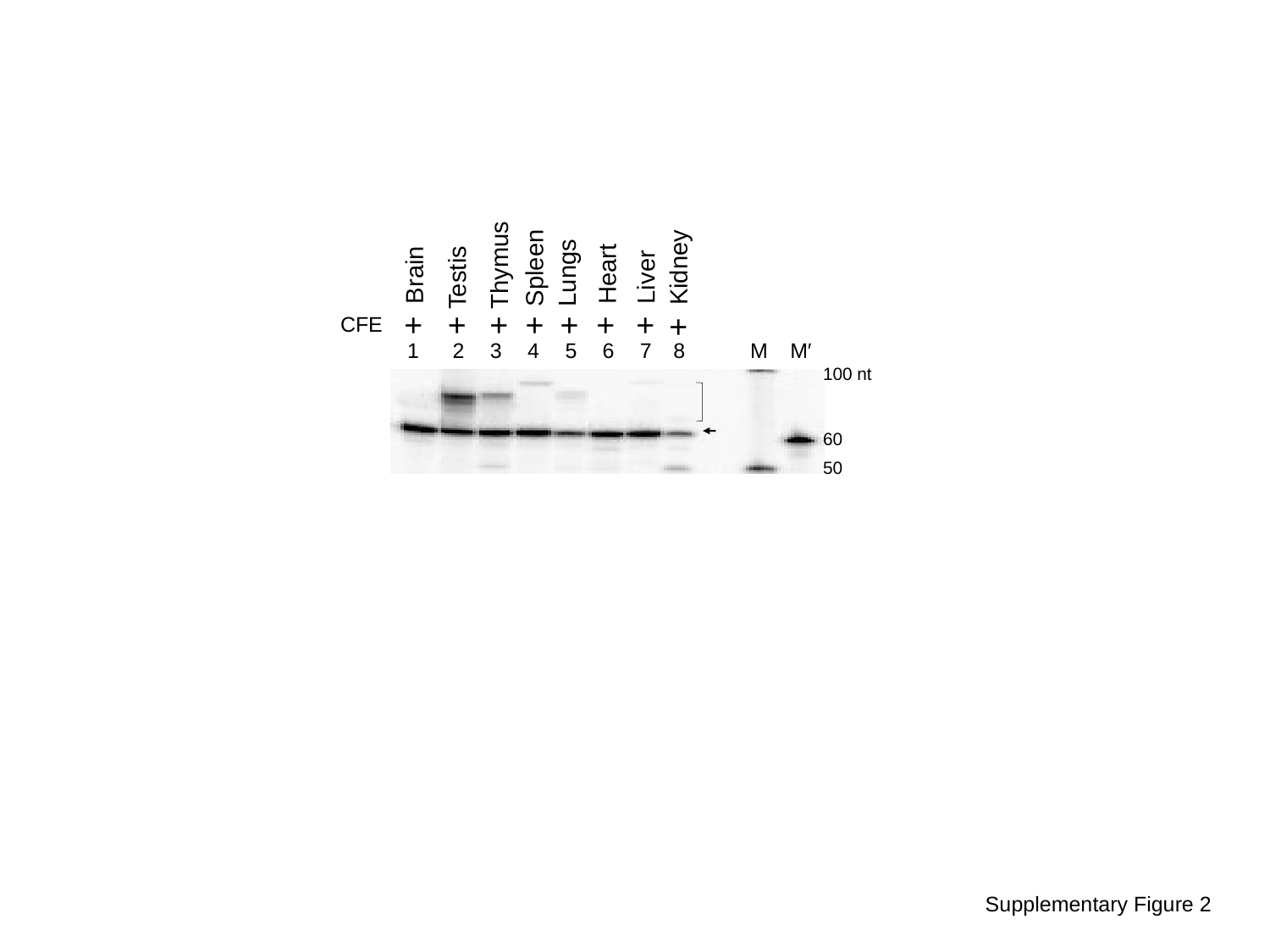

Kidney
Spleen
Lungs
Heart
Brain
Liver
Testis
+
+
+
+
+
+
+
+
CFE
1
2
3
4
5
6
7
8
M
M′
100 nt
60
50
Thymus
Supplementary Figure 2

## Slide 5
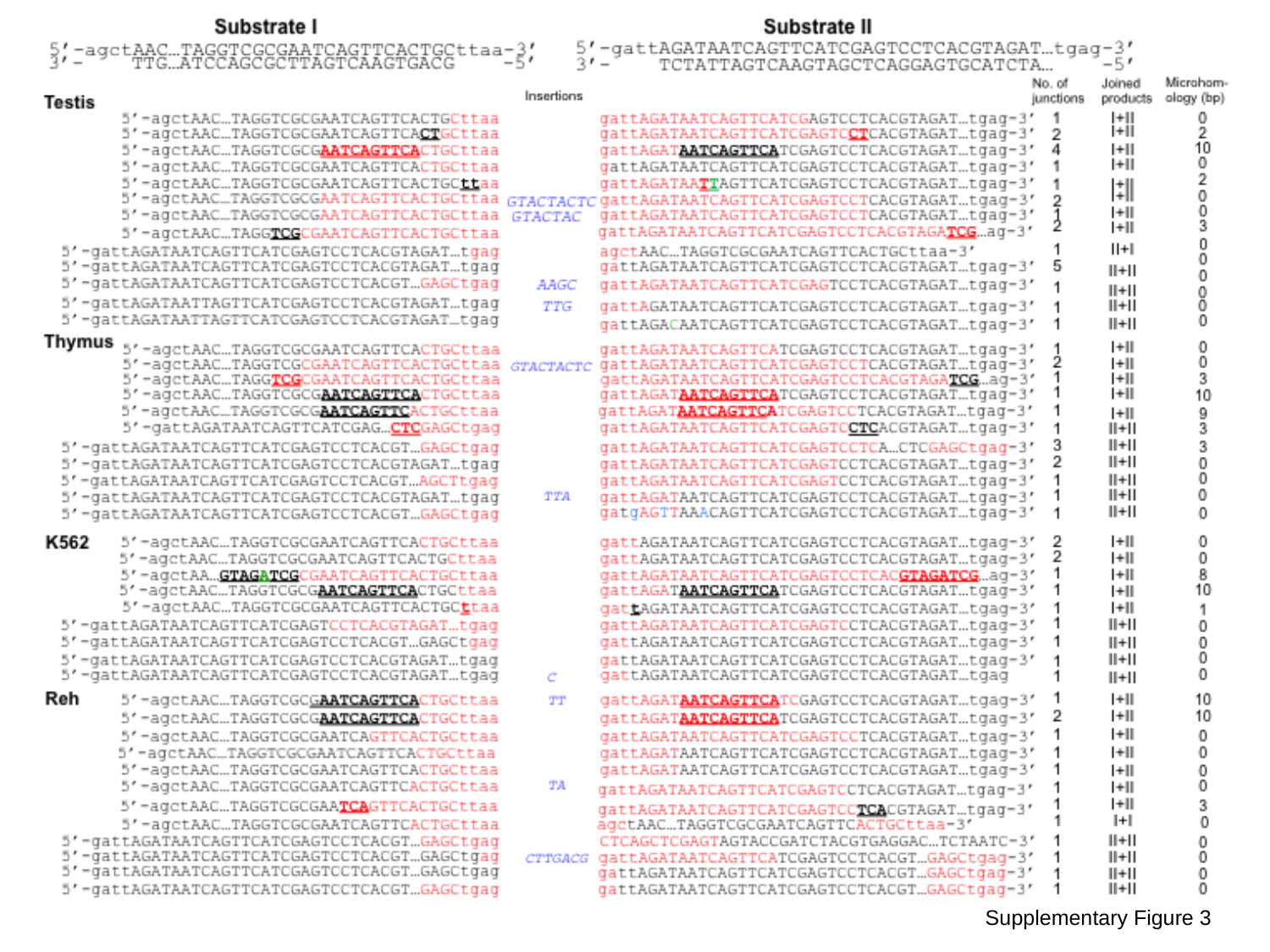

Supplementary Figure 3

## Slide 6
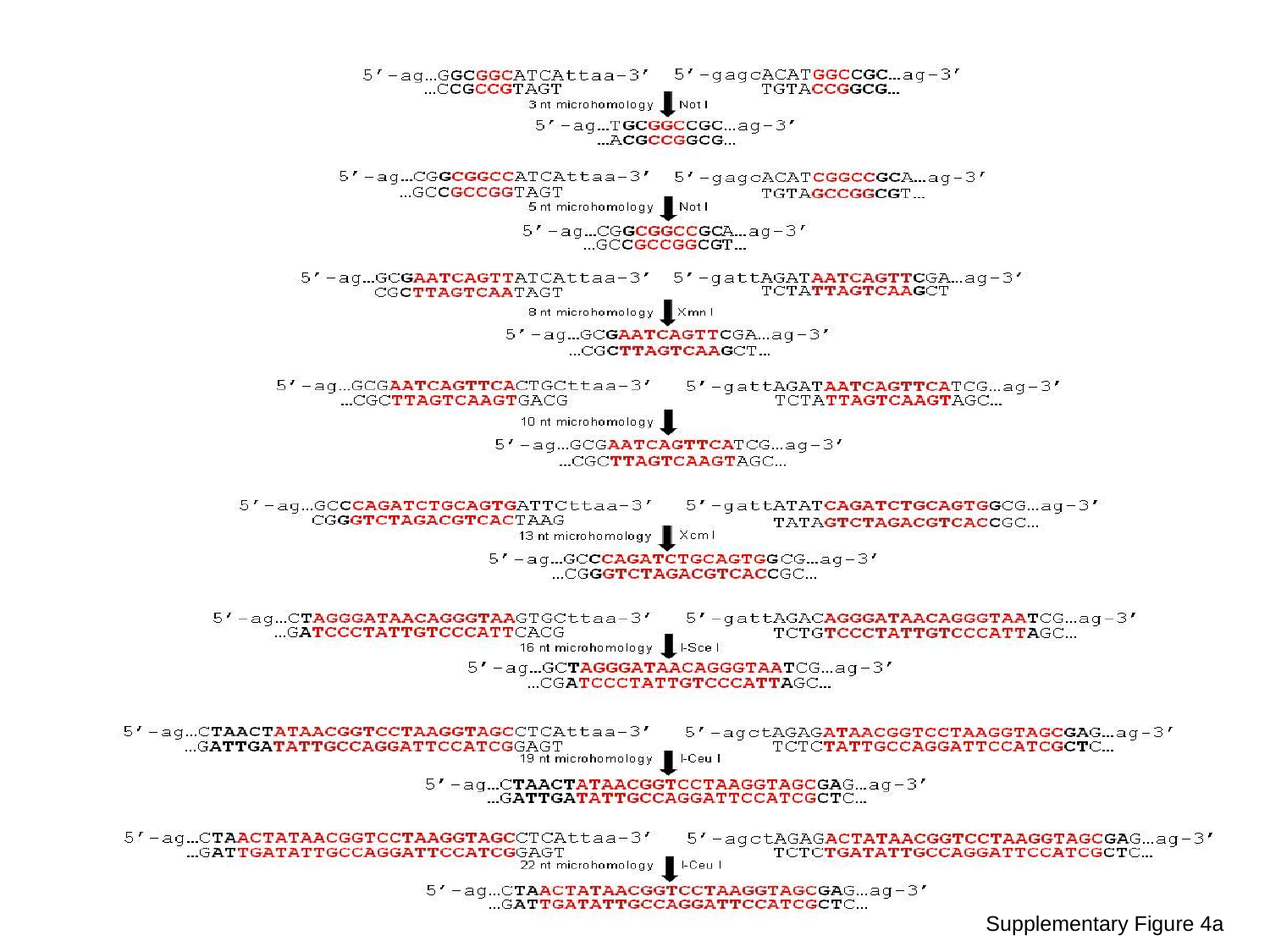

Supplementary Figure 4a

## Slide 7
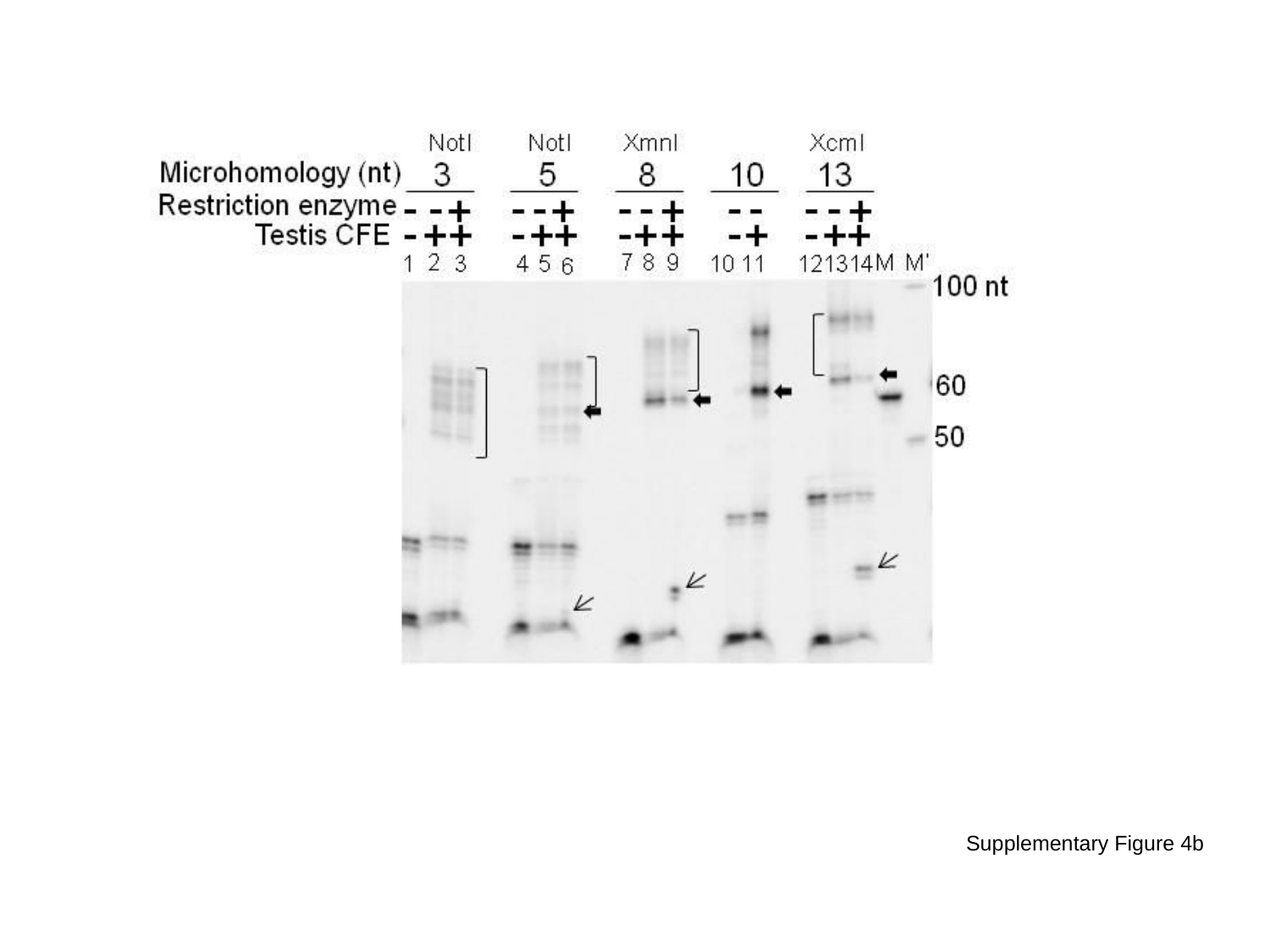

Supplementary Figure 4b

## Slide 8
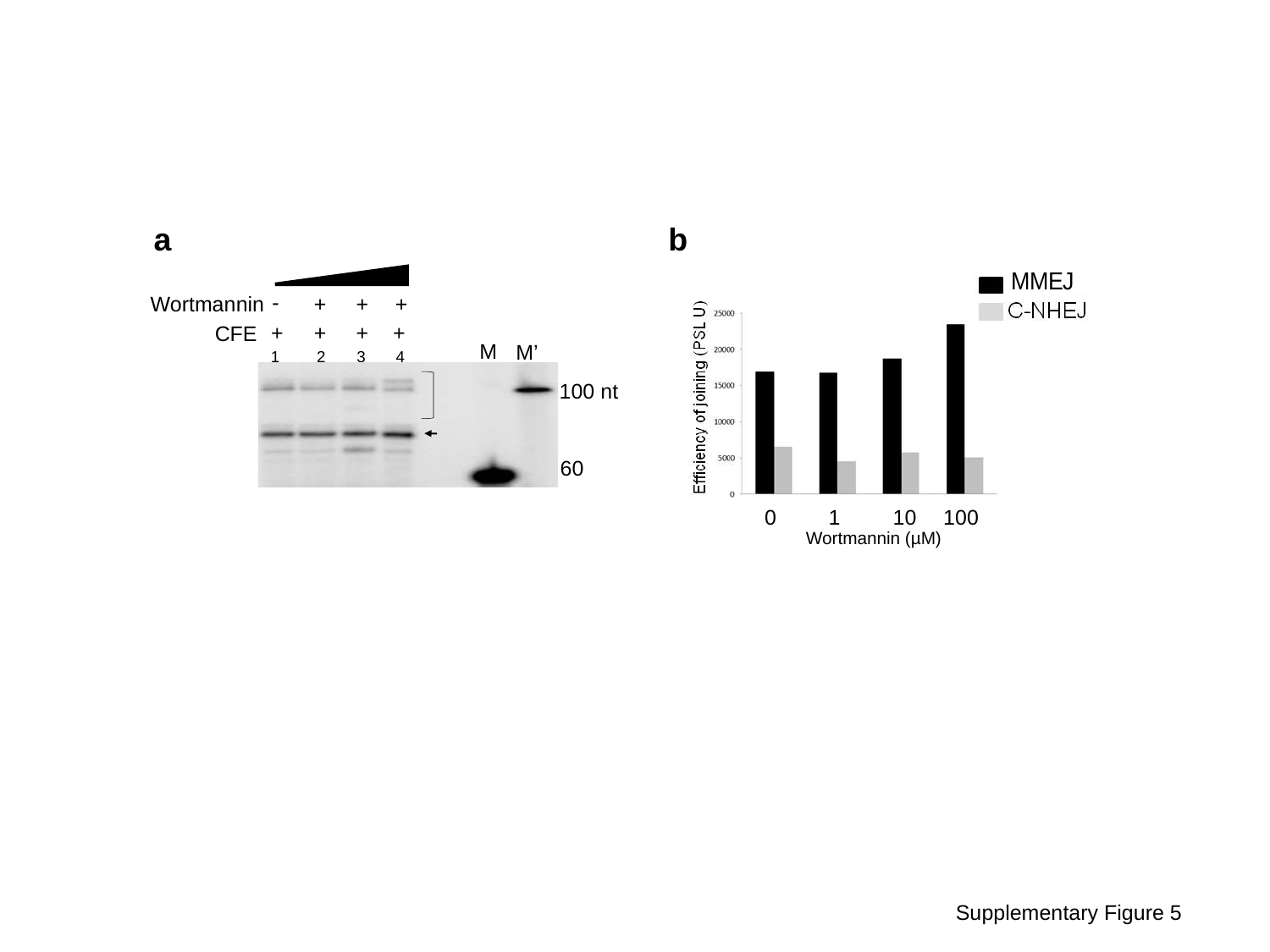

a
b
-
+
+
+
Wortmannin
+
+
+
+
CFE
M
M’
1
2
3
4
100 nt
60
0
1
10
100
Wortmannin (µM)
Supplementary Figure 5

## Slide 9
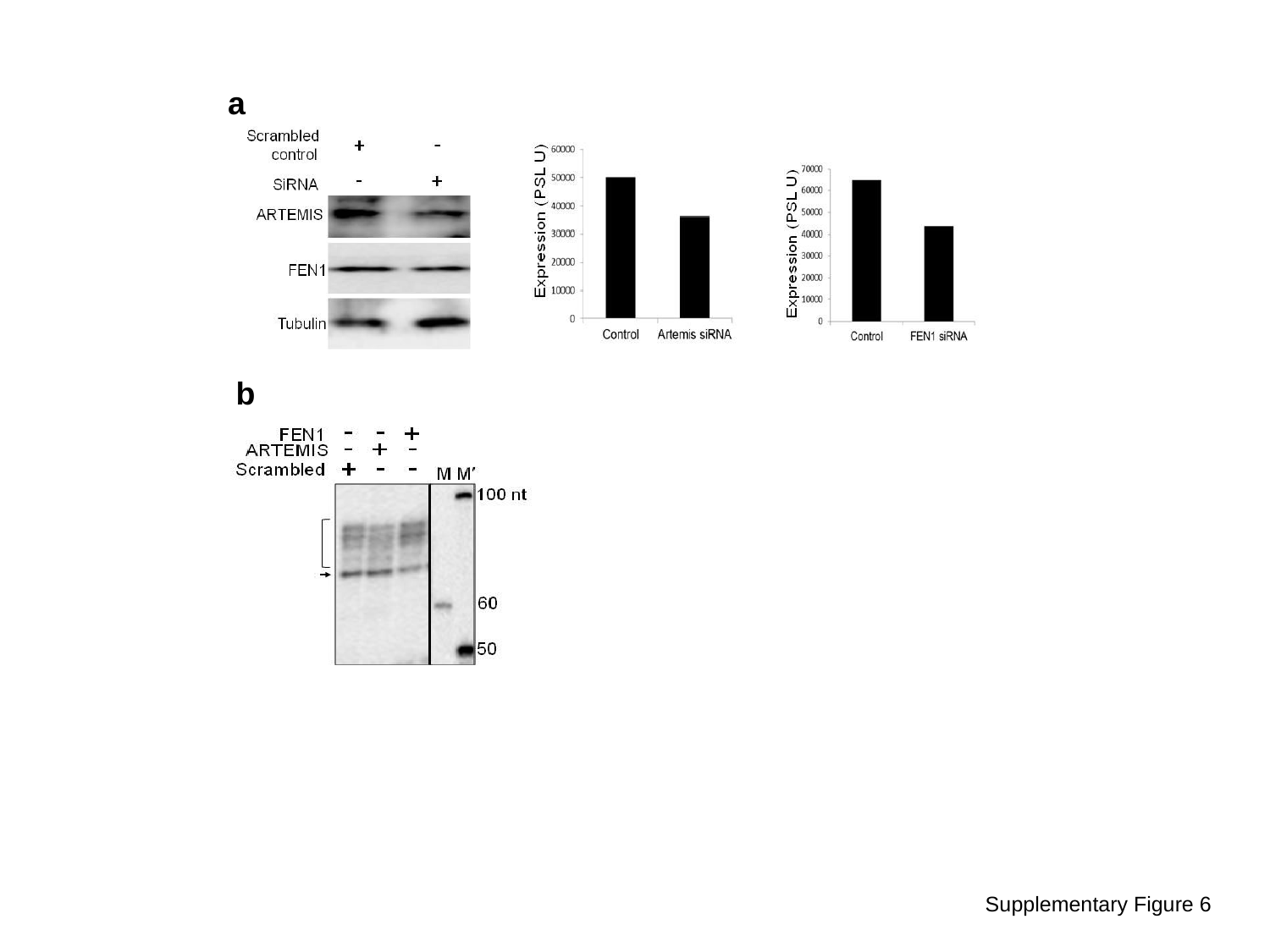

a
b
Supplementary Figure 6
